# Triple hydrogen bonding in a circular arrangement: ab initio, DFT and first-principles MD studies of *tris*-hydroxyaryl enamines

**DOI:** 10.1007/s10822-012-9597-3

**Published:** 2012-09-07

**Authors:** Agata Martyniak, Jarosław Panek, Aneta Jezierska-Mazzarello, Aleksander Filarowski

**Affiliations:** Faculty of Chemistry, University of Wrocław, 14 F. Joliot-Curie str., 50-383 Wrocław, Poland

**Keywords:** Circle hydrogen bonding formation, Schiff base, Keto-enamine, Enol-imine, Tautomeric equilibrium, CPMD, MP2, DFT, Potential energy function, Conformational analysis

## Abstract

First-principles Car-Parrinello molecular dynamics, ab initio (MP2) and density functional schemes have been used to explore the tautomeric equilibrium in three *tris*(amino(R)methylidene)cyclohexane-1,3,5-triones (R = hydrogen, methyl or phenyl group). The dynamic nature of the cyclic hydrogen bonding has been studied by the first-principles MD method. The comparison of the results obtained by aforesaid methods has been accomplished on the basis of calculations of structural and spectroscopic characteristics of the compounds. The conformational analysis of the studied compounds has been carried out at the MP2/6-31+G(d,p) and B3LYP/6-31+G(d,p) levels of theory. The influence of steric and electronic effects on the cyclic hydrogen bonding has been analysed. The extent of the proton delocalization has been modified by the substituents according to the sequence: hydrogen < phenyl < methyl. This fact is verified by the spectroscopic and structural data as well as the energy potential curve. A prevalence of the keto-enamine tautomeric form has been observed in the static ab initio and DFT models, and confirmed by the first-principles MD.

**Electronic supplementary material:**

The online version of this article (doi:10.1007/s10822-012-9597-3) contains supplementary material, which is available to authorized users.

## Introduction

The presented *tris*(amino(R)methylidene)cyclohexane-1,3,5-triones belong to the ortho-hydroxyaryl Schiff formation which is an object of wide physicochemical studies [1–3]. The great interest in ortho-hydroxyaryl Schiff bases is grounded on the application in medicine [4, 5] and the industrial chemistry [6, 7]. It is noteworthy that vitamin B6 is a heterocyclic derivative of ortho-hydroxy Schiff bases. Despite its wide application, only recently vitamin B6 has been studied and analyzed with quantum–mechanical methods [8, 9]. The combined quantum and classical mechanics calculations of vitamin B6 (pyridoxal 5′-phosphate derivative) have been carried out to elucidate the factors that contribute to the tautomeric equilibrium [8]. The latest investigations [10, 11] of two successfully modelled dimensional fluorescence switches are based on the strengthening of π-electronic conjugation in *tris*-salicylideneamine by the doubled cyclic intramolecular hydrogen bonding. This approach makes it possible to enhance a fluorescent quantum yield of these compounds which refer to the mechanically coupled biconcave systems [11]. *Tris*-salicylideneanilines with long liquid crystal substituents have been recently classified as a new class of discotic liquid crystal substances [12, 13], where a triple quasi-aromatic core is the basic fragment. The physicochemical characteristics of the aforesaid compounds enable the employment in organic electronics, optoelectronics and photovoltaics, as a molecular logic gate, photochromic crystals and reversible molecular switches [14, 15].

Though the experimental studies and synthesis of *tris*-salicylideneanilines are becoming more popular [10–13, 16, 17], quantum–mechanical calculations are very few in the literature [16, 17]. The intramolecular tautomeric equilibrium is an interesting feature of these compounds. It is useful to employ modelling molecular techniques for this kind of compounds to describe the physicochemical properties of tautomeric equilibrium. In this study a dynamic and static models based on the CPMD [18], MP2 [19] and DFT [20] frameworks are used to investigate the tautomeric equilibrium in *tris*(amino(R)methylidene)cyclohexane-1,3,5-triones. The combination of the computational models was selected to obtain a broad insight into the variables modifying internally the tautomeric equilibrium. The theoretical and experimental studies of the tautomeric equilibrium in mono-hydroxyaryl Schiff bases are described in the review papers [21, 22]. A great advantage of ortho-hydroxyaryl Schiff bases is that the intramolecular tautomeric equilibrium can be easily modified by changing acidity and basicity of the proton-donor and proton-acceptor group, correspondingly. The electron-donating and electron-withdrawing substituents act as effective tuners of the intramolecular tautomeric equilibrium (acid–base balance). However, variations of these substituents fail to evoke the extra-short intramolecular OHN hydrogen bond. The most efficient tool for the extra shortening of the length of the intramolecular hydrogen bond in ortho-hydroxyaryl Schiff bases appeared to be a steric squeezing of the bulky substitute in the imine fragment [21]. Such approach to a large extent promotes the shortening of the hydrogen bridge up to the so-called short strong hydrogen bond (SSHB) or Speakman-Hadži hydrogen bond [23, 24].

**Scheme 1:**
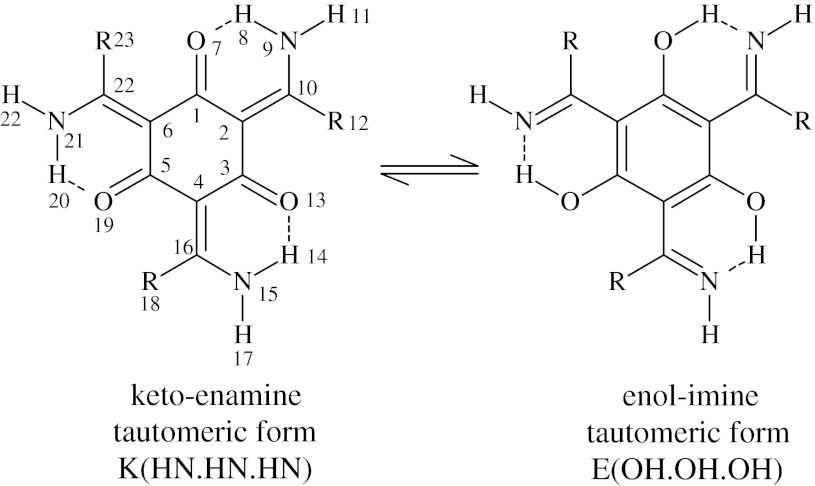
The structures of studied compounds and their tautomeric forms: (2*E*,4*E*,6*E*)-2,4,6-*tris*(1-aminoethylidene)cyclohexane-1,3,5-trione (**1**; R = CH_3_), (2*E*,4*E*,6*E*)-2,4,6-*tris*(amino(phenyl)methylidene)cyclohexane-1,3,5-trione (**2**; R = C_6_H_5_), (2*E*,4*E*,6*E*)-2,4,6-*tris*(aminomethylidene)cyclohexane-1,3,5-trione (**3**; R = H)

In this paper three subtypes of hydroxyaryl Schiff bases with three various substituents in the imine group are studied and analyzed (Scheme 1). The aldimine derivative ((2*E*,4*E*,6*E*)-2,4,6-*tris*(aminomethylidene)cyclohexane-1,3,5-trione—**3**) has no bulky substituent (R = H) and refers to (2*E*,4*E*,6*E*)-2,4,6-*tris*(1-aminoethylidene)cyclohexane-1,3,5-trione—**1** (R = CH_3_) and (2*E*,4*E*,6*E*)-2,4,6-*tris*(amino(phenyl)methylidene)cyclohexane-1,3,5-trione—**2** (R = C_6_H_5_) which carry the methyl and phenyl groups, correspondingly.

It is a complicated task to calculate the intramolecular interrelations and the static methods employed are not always successful. Moreover, the description of the ensembles with hydrogen bonds and media with prototropic interrelations takes much effort as well. So, the progress in computational techniques is accompanied with the development of new advanced methods of quantum dynamics rested upon the combination of both classical and quantum mechanics [25–27]. The CPMD method enables a dynamical description of hydrogen bond interactions between molecules [18, 25, 28, 29]. It should be noted that the studies of the molecules with intramolecular hydrogen bonds by the CPMD method have advanced recently [30–34]. The paper also puts an objective to compare the performance of the static DFT and MP2 calculations with the CPMD dynamic method for the description of the tautomeric equilibrium.

## Computational methodology

### Car-Parrinello molecular dynamics

The dynamical nature of the investigated molecules **1**–**3** (Scheme 1), with emphasis on their hydrogen bridges, was studied using the Car-Parrinello molecular dynamics (CPMD) methodology [35]. This technique, based on the Density Functional Theory (DFT), provides a good framework for the investigation of dynamical properties in situations beyond the applicability of classical force fields. The examples of necessity of using ab initio molecular dynamics schemes, including CPMD, involve the bond breaking and forming processes and detailed studies of hydrogen bonds. The CPMD calculations were carried out in the gas phase, with the Perdew–Burke–Ernzerhof (PBE) exchange–correlation DFT functional [36]. Core electrons of the studied molecules were replaced by norm-conserving pseudopotentials of the Troullier-Martins type [37], while the Kohn–Sham orbitals were expanded using the plane-wave basis set with the maximum kinetic energy cutoff of 100 Ry. This cutoff for the plane wave expansion must be sufficient for the norm-conserving pseudopotential scheme, and we tested convergence of the energy and forces with respect to the cutoff. The molecular structures were placed in a cubic box with a = 18 Å for **1** and **3**, 24 Å for **2**, which leaves ample space between the box walls and the electronic density of the molecule, required by the Hockney’s scheme [38] and used to remove interactions with periodic images and simulate isolated molecule conditions. In the preliminary step, structures of compounds **1**–**3** were optimized with the initial Hessian function constructed using Schlegel’s scheme [39]. Then, the CPMD runs were carried out. The orbital coefficients were propagated using a default value of the fictitious orbital mass, 400 a.u., and the nuclear motion timestep was set to 3 a.u. (0.0726 ps), short enough to represent accurately fast motions of the hydrogen atoms and associated changes in the orbital coefficients. The ionic temperature was set to 298 K and controlled by Nosé-Hoover thermostat chains with default settings, each degree of freedom coupled to a separate thermostat (“massive” thermostating) [40, 41]. The electronic degrees of freedom were also coupled to a Nosé-Hoover thermostat chain set to 15,000 cm^−1^ frequency. The data collection, after the initial equilibration phase, lasted ca. 15 ps for compound **1** and 10 ps for compounds **2** and **3**. The obtained trajectories served as a basis for the distance evolution analysis and determination of vibrational features from the power spectra of atomic velocity.

### Free energy profile for proton motion on the basis of the blue-moon ensemble

The statistical sampling provided by molecular dynamics schemes allow to calculate the free energy profile for proton motion in the intramolecular hydrogen bridge. The constrained MD scheme based on the blue-moon ensemble was applied to calculate the free energy profiles [42]. The constrained variable was the NH distance in each hydrogen bridge. The values of the variable were gradually changing from 0.9 to 2.0 Å. For each fixed NH distance, a short CPMD run (1.5 ps) was carried out using the setup described in the previous paragraph. The constraint force was collected during each run. The first half of each run was taken as initial equilibration phase, and then the second half was used subsequently for statistical averaging. The averaged constraint forces were used to obtain the free energy profile (potential of mean force, pmf) [42] by numerical integration.

The Car-Parrinello MD simulations were carried out using the CPMD 3.11.1 program [43], and the data analysis was performed using locally written utilities. The graphs were prepared with the Gnuplot graphics package [44], and the visualizations were created with the VMD 1.8.6. program [45].

### Static ab initio and DFT calculations

**Scheme 2:**
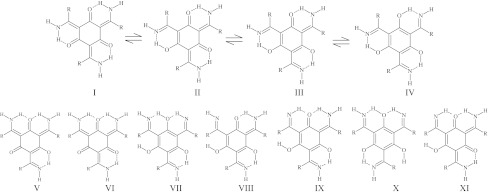
Tautomeric and conformational scheme of the studied compounds

This part of calculations was performed with the Gaussian 09 [46] suite of programs. Double zeta split valence basis set denoted as 6-31+G(d,p) [47–50] according to the Pople’s notation was applied. The static models were developed on the basis of density functional theory using the three parameter functional proposed by Becke with correlation energy according to the Lee–Yang–Parr formula, denoted as B3LYP [51, 52] and second-order perturbative Møller–Plesset (MP2) methods [19, 53]. The use of diffuse functions is a proper approach for studies of hydrogen bonding [54]. Initially, structural optimization was carried out and followed by harmonic frequencies calculations confirming that the obtained structures correspond to the minima on the potential energy surface. The conformers and tautomers were prepared according to Scheme 2. Further, the energy of conformers and tautomers was calculated and analysed using the reference structures presented in Scheme 1. The reaction path of the hydrogen atom involved in the intramolecular hydrogen bond formation was studied. One-dimensional approach was employed to analyse in detail the proton reaction path. The applied approach is based on stepwise elongation of the NH bond length (with 0.1 Å increment) with full optimisation of the remaining structural parameters. This approach reflects the proton transfer process in the intramolecular hydrogen bridge.

## Results and discussion

### The estimation of the tautomeric equilibrium by means of first principles molecular dynamics (CPMD), and static (ab initio and DFT) quantum–mechanical methods

**Fig. 1:**
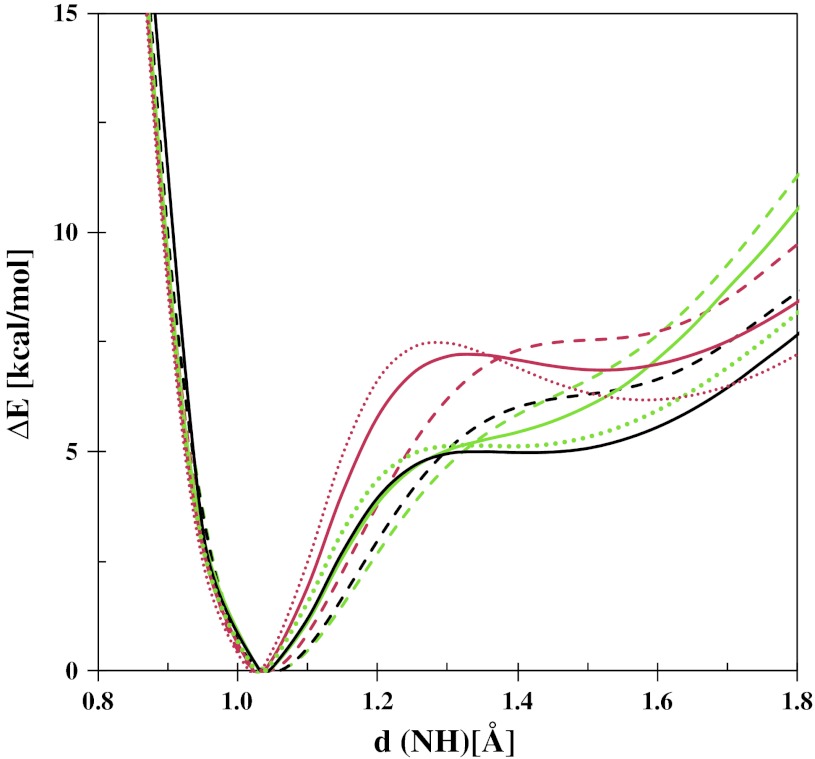
Calculated (*dotted line* MP2/6-31+G(d,p), *solid line* B3LYP/6-31+G(d,p), *dashed line* CPMD) potential energy functions for the gradual proton displacement in the intramolecular hydrogen bond of **1**–**3**. The *colour* coding of the curves is: *green* for compound **1**, *black* for compound **2** and *red* for compound **3**. Results of the constrained Car-Parrinello MD based on the blue-moon ensemble method

**Fig. 2:**
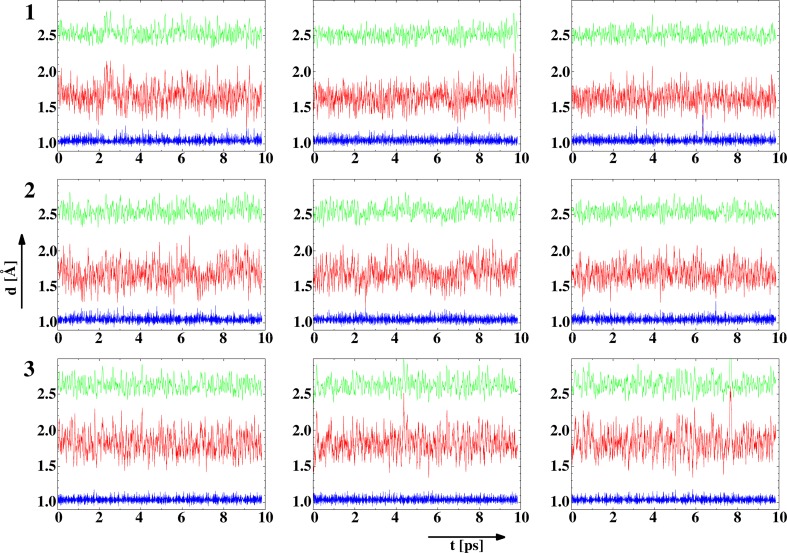
Time evolution of the O···N, O–H and H–N interatomic distances for the hydrogen bonds in compounds **1**–**3**—results of the CPMD simulation at the gas phase. The *colour* coding of the graphs is: *green* the d(ON) distance, Å; *red* the d(OH) distance, Å; *blue* the d(OH) distance, Å. *Each row* represents data for the three intramolecular bridges of one compound indicated on the *left*

The collation of experimental and theoretical results [55–57] predicts the behaviour of the tautomeric equilibrium under the experimental conditions on the basis of potential curves calculated with non-adiabatic approximations. Thereby, for the estimation of the tautomeric equilibrium state the potential curves have been calculated for the studied compounds. The potential curves (Fig. 1) calculated by the DFT and MP2 methods are similar which points out the prevalence of the keto-enamine tautomeric form (the global minimum is observed under the d(NH) ≅ 1 Å; d(OH) ≅ 1.7 Å). It is noticeable that the completed calculations show that the keto-enamine tautomeric form is more likely to be detected (under the calculated conditions: T = 0 K, gas phase) than the enol-imine tautomeric form (the local minimum is observed under the d(NH) ≅ 1.7 Å; d(OH) ≅ 1 Å). The obtained energy difference is not large enough (ΔE_OH–NH_ = 4–6 kcal/mol) to prevent the presence of the enol-imine tautomeric form under the experimental conditions. To overcome the difficulties of describing the dynamical behaviour of a strongly modulated bridge, the potential of mean force (pmf) was calculated for the proton motion, which corresponds to the free energy profile. Car-Parinello molecular dynamics (CPMD) simulations were performed in vacuum for the investigated compounds. Regardless of the tautomeric form optimized at the PBE/plane-wave level, the gas-phase CPMD runs led to instantaneously the protons transfer to the nitrogen atoms, showing the prevalence of the keto-enamine form. The simulations indicate that the protons were localized on the nitrogen atoms at the gas phase (Fig. 2).

The shapes of these potential curves calculated by means of the CPMD method also verify the prevalence of the keto-enamine tautomeric form (Fig. 1). However, these potential curves are more gently sloping than those obtained by static methods and the local minimum (corresponding to the enol-imine tautomeric form) is not observed. This phenomenon is provoked by a strong dynamics of protons in the hydrogen bridges and there is no possibility to trace the local minimum under d(NH) ≅ 1.5–1.6 Å by the CPMD method. However, the minimum of the potential curve at the nitrogen atom is wider, and enables a large amplitude motions of the proton. This effect corresponds to the broadening of the vibrational band which is observed in the infrared spectra.

**Fig. 3:**
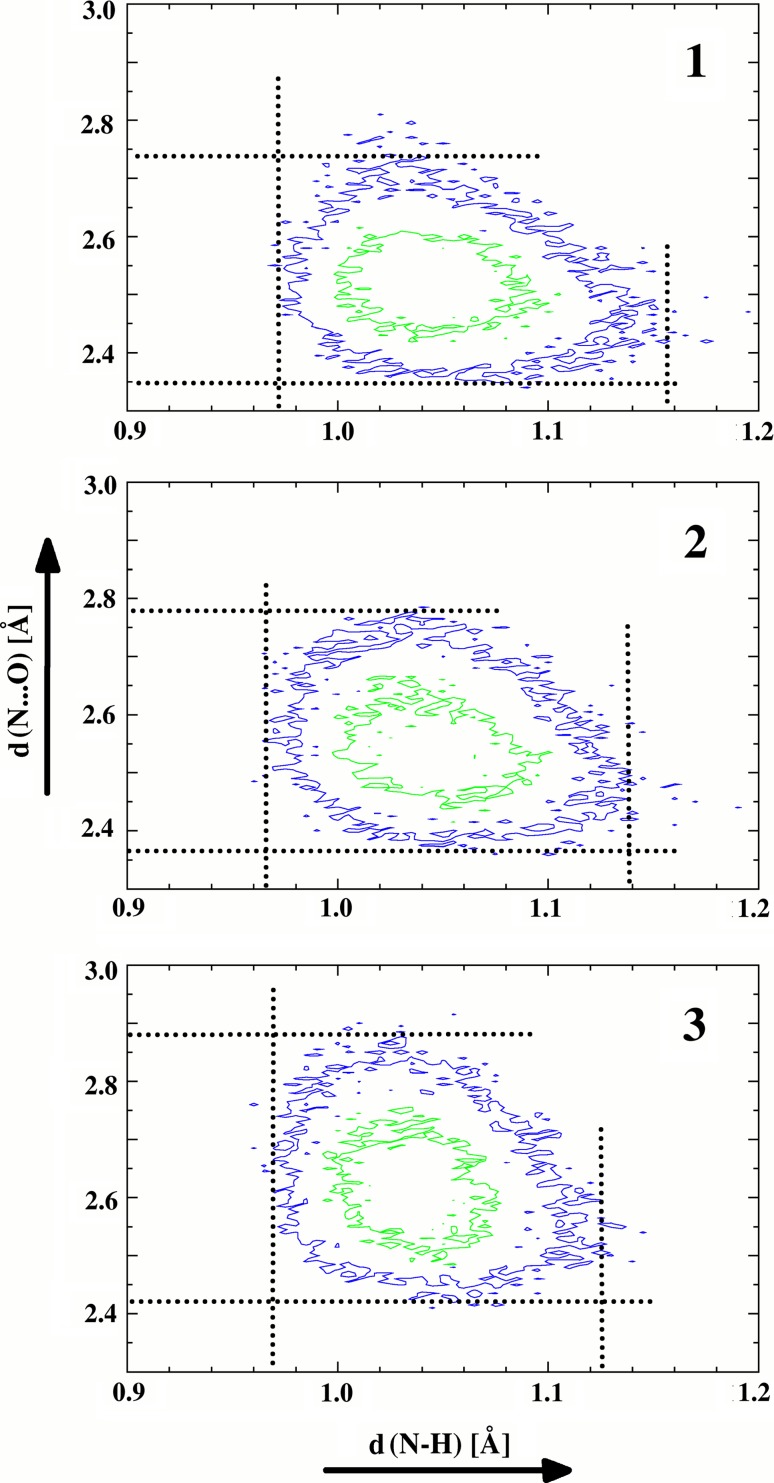
Separate histograms of proton detection obtained from the CPMD trajectory as function of the donor-proton (d(N···O)) and donor–acceptor (d(N–H)) distances for compounds **1**–**3** (**1**—∆d(N–H) = 0.972–1.158 Å and ∆d(N···O) = 2.35–2.74 Å; **2**—∆d(N–H) = 0.965–1.139 Å and ∆d(N···O) = 2.37–2.78 Å; **3**—∆d(N–H) = 0.968–1.125 Å and ∆d(N···O) = 2.42–2.88 Å). The proton detection contours are drawn at 5 Å^−2^ (*blue*) and 30 Å^−2^ (*green*) levels

One of the most important advantages of the CPMD method is the capability to estimate both the mobility of the bridged hydrogen and the dynamics of the hydrogen bonding. To define these characteristics the CPMD calculations were carried out, which are presented as separate histograms (the distance of proton-donor–proton-acceptor vs the distance donor-hydrogen, Fig. 3). These histograms show that the hydrogen mobility in the hydrogen bridge is increasing with the strengthening of the hydrogen bond [the reduction of the hydrogen bridge length, d(ON)]. The span of maximum probable detection of the bridged proton is growing according to the **3** < **2** < **1** sequence (Δd(NH) = d_max_(NH) − d_min_(NH); **1**—Δd(NH) = 1.158 − 0.972 = 0.186 Å; **2**—Δd(NH) = 1.139 − 0.965 = 0.174 Å; **3**—Δd(NH) = 1.125 − 0.968 = 0.157 Å). The forms of these histograms also defined a larger mobility of the bridged hydrogen in compounds **1** and **2** than in compound **3**. For compound **3** this form takes a round shape (which is the confirmation of the proton localization), whereas it tends to be more oval for compounds **1** and **2** (which speaks in favour of a larger delocalization of proton in the hydrogen bridge).

On the other hand, the reverse dependence is observed for the hydrogen bridge length. The strengthening of the hydrogen bonding (Table 1) brings about a decrease of the amplitude of the vibrations of the hydrogen bridge (Δd(NO) = d_max_(NO) − d_min_(NO); **1**−Δd(NO) = 2.74 − 2.35 = 0.39 Å; **2**—Δd(NO) = 2.78 − 2.37 = 0.41 Å; **3**—Δd(NO) = 2.88 − 2.42 = 0.46 Å). It is important to point out that this trend can be explained by a significant steric squeezing of the bulky (methyl or phenyl) group, which prevents the hydrogen bridge vibrations amplitude from growing. Moreover, the comparison of the data collected in Refs. [58, 59] makes it possible to generalize that the decrease of the hydrogen bridge vibrations amplitude under the bridge strengthening is a common phenomenon for the intramolecular hydrogen bonding with the prevailing of one of the tautomeric forms.

**Table 1:** Calculated (DFT—B3LYP/6-31+(d,p); *CPMD*—Car-Parrinello molecular dynamics) center of gravity of stretching mode (in cm^−1^) and energy (kcal/mol) of hydrogen bonding by ∆E_HB_ = (−5.554 × 10^5^) × exp(−4.12 × r(ON)) [65] equation for the studied compounds

Compound	**1**			**2**			**3**		
Tautomeric form	E(OH·OH·OH)	K(NH·NH·NH)		E(OH·OH·OH)	K(NH·NH·NH)		E(OH·OH·OH)	K(NH·NH·NH)	
Method	DFT	DFT	CPMD	DFT	DFT	CPMD	DFT	DFT	CPMD
ν(OH)1	1,948.2	–	–	2,448.4	–	–	2,668.4	–	–
ν(OH)2	1,967.1	–		2,459.9	–		2,686.2	–	–
ν(OH)3	1,973.8	–		2,463.3	–		2,686.5	–	–
ν(HN)1	–	3,225.7	2,870	–	3,265.4	2,950	–	3,406.1	3,105
ν(HN)2	–	3,232.7		–	3,269.4		–	3,409.1	
ν(HN)3	–	3,236.0		–	3,270.4		–	3,410.6	
ΔE_HB_	27.29	17.71	18.99	21.12	15.51	17.03	16.49	10.44	12.09
ΔE_HB_	27.00	17.75	18.01	21.14	15.56	17.75	16.60	10.45	12.21
ΔE_HB_	26.94	17.57	18.74	21.05	15.52	17.65	16.51	10.42	12.21

**Scheme 3:**
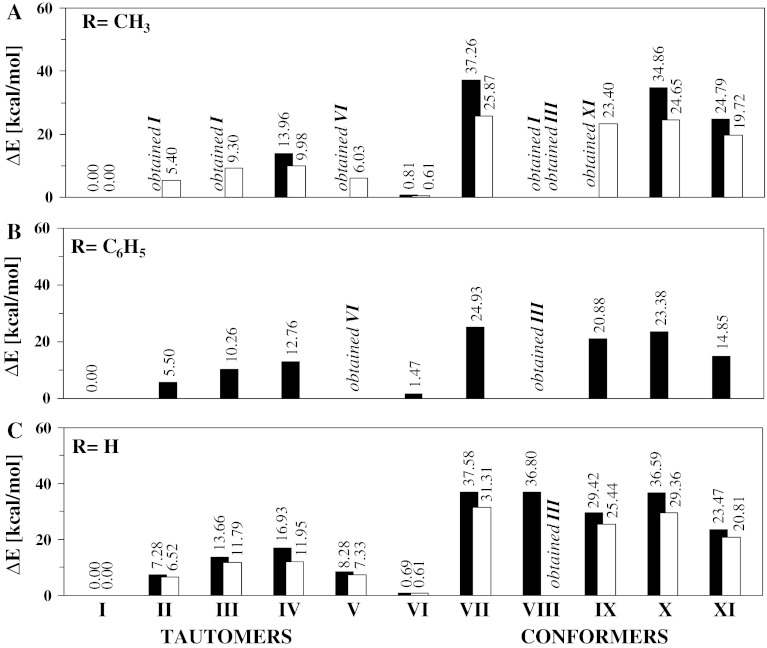
Energy scheme of tautomers and conformers for the **1** (**a**), **2** (**b**) and **3** (**c**) compounds calculated at the B3LYP/6-31+G(d,p) (*black columns*) and MP2/6-31+G(d,p) (*white columns*) levels of theory. The conformers of compound **2** are not obtained at the MP2/6-31+G(d,p) levels of theory due to equilibrium of the phenyl groups during the structure optimization process

To define the prevalence of a particular conformer of a tautomeric form in the compounds with cyclic hydrogen bonds the conformational analysis was also carried out at the B3LYP/6-31+G(d,p) and MP2/6-31+G(d,p) levels of theory. This conformational analysis rests upon the full geometry optimization of the molecule for various possible conformational structures (Scheme 2). All possible combinations of protons in the three hydrogen bridges have also been calculated. This gives a deeper insight into the hydrogen bridge reorganization upon the proton displacement. The obtained energetic characteristics (Scheme 3) show that the proton transfer in compound **3** results in the increase of ΔE_PT_ (ΔE_PT_ = E_NH_ − E_OH_). Every next proton transfer [the transition from tautomeric form I to tautomeric form IV (I → II → III → IV)] calls forth the shortening of the hydrogen bridge length (Tables 1S and 2S) and, consequently, the strengthening of the hydrogen bonding. Three step-by-step processes of the protons transfer requires some energy expenditure: ΔE_PT_ = 7.28 → 13.66 → 16.93 and 6.52 → 11.79 → 11.92 kcal/mol calculated at the B3LYP/6-31+G(d,p) and MP2/6-31+G(d,p) levels of theory, respectively (Scheme 3). The obtained ΔE_PT_ value (particularly 7.28/6.52 kcal/mol; B3LYP/MP2) is not large and two enol-imine and keto-enamine tautomeric forms could be observed under experimental conditions. The energy barrier (ΔE_TS_ = E_OH_ − E_TS_), which is a more relevant parameter than the ΔE_PT_ for the description of the protons transfer process, was also calculated. However, the calculated ΔE_TS_ values show a large energy barrier (ΔE_TS_(TS, TS, TS) = 20.62 kcal/mol → ΔE_TS_(OH, TS, TS) = 19.45 kcal/mol → ΔE_TS_(OH, OH, TS) = 18.45 kcal/mol (B3LYP/6-31+G(d,p)) and don’t support the observation of the enol-imine tautomeric form under experimental conditions. This conclusion is consistent with the experimental results published by MacLachlan [16, 17], Lee [10, 11], Yelamaggad [12, 13] and Suresh [60], which also confirm the prevalence of the keto-enamine tautomeric form. For the studied compounds a number of conformers was also obtained (VII–XI), all of them possessing a much larger energy (ΔE > 20 kcal/mol) than the energy of the most stable conformer I. This fact puts in question the detection of these conformers by experimental methods. However, the energy of conformer VI is only 0.6–1.5 kcal/mol larger than the energy of the most stable conformer I, so this suggests a possibility of the conformer detection experimentally. Remarkably, the confirmation of this supposition, predicted by the theoretical calculations, was found first by MacLachlan [17] for *tris*(*N*-salicylideneamines)s. On the basis of ^1^H–^1^H Cosy NMR spectra the presence of two conformers I and VI of *tris*(*N*-salicylideneamines)s was stated in the different solvents [17]. Subsequently, similar trends are observed for compounds **1** and **2**. Likewise, some conformers of compounds **1** and **2** were not detected by the MP2 or DFT methods. However, it is remarkable that both MP2 and DFT methods complement each other and present a common conformational scheme (Scheme 3).

### The influence of the substitutions on spectral and structural characteristics of the hydrogen bridges

**Fig. 4:**
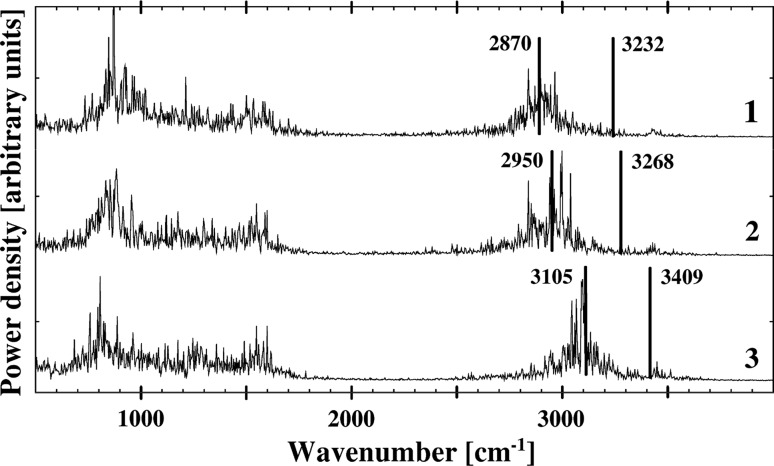
Atomic velocity power spectra for the protons in the intramolecular hydrogen bonds of the studied compounds. The spectra presented only for the bridged protons vibrational modes. 2,870 cm^−1^ (**1**), 2,950 cm^−1^ (**2**) and 3,105 cm^−1^ (**3**) center of gravity bands are obtained by CPMD method. 3,232 cm^−1^ (**1**), 3,268 cm^−1^ (**2**) and 3,409 cm^−1^ (**3**) bands positions are obtained at the B3LYP/6-31+G(d,p) level of theory

The results of the CPMD calculations show a shift of the band within the region ~3,000 cm^−1^ to the direction of low frequencies region according to the **3** < **2** < **1** sequence (Fig. 4). This band is assigned to stretching vibrations (ν(NH)) of the bridged proton due to the localization of the protons close to the nitrogen atoms. This sequence is also supported by the B3LYP/6-31+G(d,p) calculations (ν(NH) = 3,225–3,236 cm^−1^ (**1**); 3,265–3,270 cm^−1^ (**2**); 3,405–3,410 cm^−1^ (**3**), Table 1 and Fig. 4). These trends point out the strengthening of the hydrogen bonding according to the **3** < **2** < **1** sequence which was found experimentally for mono hydroxyaryl Schiff bases [61–63]. The result is also consistent with the calculated structural and energetic data (Tables 1 and 1S). The hydrogen bond distance is getting longer according to the **1** < **2** < **3** sequence for keto-enamine and enol-imine forms (Tables 1, 1S and 2S) and, thus, the hydrogen bonding is strengthening according to the **3** < **2** < **1** sequence. A similar tendency is stated for the energy of the hydrogen bonding calculated with the formula ΔE_HB_ = (−5.554 × 10^5^) × exp(−4.12 × d(ON)) [64]. The results obtained by two different methods (static—ΔE_HB_ and dynamic—ν(NH), Table 1) are fully consistent. This picture can be explained by electronic and steric effects of the phenyl and methyl groups substituted in the imine group. The electronic effects of the phenyl group is supposed to attenuate the basicity of the nitrogen atom with reference to the hydrogen substituent [65]. The phenyl group might weaken the hydrogen bonding and, therefore, elongate the hydrogen bridge. Nevertheless, the steric squeezing of the phenyl group is powerful and evokes the reverse effect—the reduction of the hydrogen bridge length and the consequent strengthening of the hydrogen bonding in compound **2**. As for the methyl group influence it is manifested through the co-directed action of steric and electronic effects which makes the hydrogen bond in compound **1** stronger than in compounds **2** and **3**.

The correlations between motions of the protons in the bridges were also analyzed on the basis of the CPMD trajectories. A large mobility of the bridged protons leads to several events of equalization between the donor-proton and proton-acceptor distances, which are rather rare and short-lived (Fig. 2). The details of these events (Figure 1S, Supporting Information) show that the fast N–H vibrations of the protons are less correlated than the slower O···H vibrations. The short-lived equalization of the d(NH) and d(OH) distances in one bridge is followed after some time by a similar event in one of the other bridges. This supports the notion of cooperativity between the bridges, remarked earlier on the basis of the proton transfer barriers. However, this cooperativity is not strongly marked.

As it was shown above, the first principles molecular dynamics and the classical DFT method provided agreeing trends. The CPMD method was applied to obtain broad bands conditioned by statistical averaging of the degrees of freedom of the molecules. Remarkably, the obtained broadening of the band is in accordance with the principles elaborated in the IR spectroscopy [66]. Meanwhile, the results of the “static” calculations determine only the center of the band. Therefore, it is advantageous to provide an insight into spectroscopic assignments also with the CPMD method.

## Conclusion

The main aim of the current studies was the investigation of an unusual system of three hydrogen bridges arranged in a circular form, and conjugated by a common aromatic system. For this purpose, the tautomeric equilibrium in the *tris*(amino(R)methylidene)cyclohexane-1,3,5-triones with cyclic hydrogen bonding was studied using the combination of quantum–mechanical methods providing dynamical and static models. The evident prevailing of the keto-enamine tautomeric form in the compounds under study is reproduced at the different levels of theory. In terms of the potential energy curves obtained by the MP2 and B3LYP methods, they reveal the existence of the local minimum (the enol-imine form presence). However, a thorough conformational analysis, carried out by the same methods, exhibits a significant energetic barrier for the proton transfer. The reproduction of the proton dynamics based on the CPMD approach did not show the presence of the enol-imine form. The time evolution of the interatomic distances showed a large mobility of the bridged protons. However, the events of equalization between the proton-donor–proton distance and the proton–proton-acceptor distance were rare and short-lived. Moreover, no local minima on the free energy curves were detected by the constrained CPMD simulation.

The influence of the substituents in the imine group on the hydrogen bonding was analysed on the basis of the calculated structural and spectral data. These data exposed the predominant impact of the steric effect on the hydrogen bonding. The CPMD trajectory was post-processed to reveal the spectral bands related to the bridged protons, and the substituent influence was indeed notable. This fact and also the increased band widths conform well with the sequences of energy and vibrational characteristics provided by the static models.

To sum it up, the results obtained by the dynamic and static methods led to the consequent conclusions and allowed to properly describe physicochemical properties of this group of molecules.

The energy barriers associated with the consecutive protons transfer events decrease, which indicates a degree of cooperativity between the three hydrogen bridges. This hypothesis is consistent with the CPMD results, which show the sequence of several events when a short-lived equalization of the donor-proton and proton-acceptor distances can induce a delayed event of the same kind in one of the other bridges. These facts show that the investigated systems are interesting models for the further search of tunable molecular “proton relays” in the chemical subspace of structures with multiple conjugated hydrogen bridges.

## Electronic supplementary material

Below is the link to the electronic supplementary material.

Supplementary material 1 (DOCX 110 kb)
