# Supplementary material for: Triple hydrogen bonding in a circular arrangement: ab initio, DFT and first-principles MD studies of tris-hydroxyaryl enamines
Source: J Comput Aided Mol Des. 2012 Sep 7;26(9):1045–53. doi: 10.1007/s10822-012-9597-3 (PMC3474916; doi:10.1007/s10822-012-9597-3)
Supplement: Supplementary file 1 — Supplementary material 1 (DOCX 110 kb) [file 10822_2012_9597_MOESM1_ESM.docx]

**Supplementary material**

**Table 1S** Calculated (DFT - B3LYP/6-31+(d,p); MP2 - MP2/6-31+(d,p); CPMD – Car-Parrinello molecular dynamics) structural parameters (in Å) of the hydrogen bond presented in the studied compounds

| Compound | 1 | | | | | 2 | | | | | 3 | | | | |
| --- | --- | --- | --- | --- | --- | --- | --- | --- | --- | --- | --- | --- | --- | --- | --- |
| Tautomeric form | E(OH.OH.OH) * | | K(HN.HN.HN) | | | E(OH.OH.OH) | | K(HN.HN.HN) | | | E(OH.OH.OH) | | K(HN.HN.HN) | | |
| Method | DFT | MP2 | DFT | MP2 | CPMD | DFT | MP2 | DFT | MP2 | CPMD | DFT | MP2 | DFT | MP2 | CPMD |
| Distances |  |  |  |  |  |  |  |  |  |  |  |  |  |  |  |
| OH8 | 1.0826 | 1.0252 | 1.6634 | 1.6314 | 1.5976 | 1.0373 | 0.9965 | 1.7096 | 1.7298 | 1.6433 | 1.0249 | 1.0020 | 1.8646 | 1.8691 | 1.7794 |
| OH16 | 1.0789 | 1.0212 | 1.6634 | 1.6314 | 1.6134 | 1.0376 | 0.9943 | 1.7087 | 1.7069 | 1.6330 | 1.0249 | 1.0080 | 1.8644 | 1.8691 | 1.7756 |
| OH23 | 1.0791 | 1.0240 | 1.6648 | 1.6318 | 1.6019 | 1.0372 | 1.0039 | 1.7095 | 1.7239 | 1.6333 | 1.0244 | 1.0080 | 1.8657 | 1.8692 | 1.7752 |
| HN9 | 1.3955 | 1.5288 | 1.0316 | 1.0373 | 1.0513 | 1.5121 | 1.6325 | 1.0300 | 1.0128 | 1.0470 | 1.5922 | 1.6466 | 1.0228 | 1.0183 | 1.0368 |
| HN17 | 1.4020 | 1.5472 | 1.0315 | 1.0373 | 1.0503 | 1.5115 | 1.6367 | 1.0299 | 1.0117 | 1.0475 | 1.5902 | 1.6463 | 1.0229 | 1.0183 | 1.0370 |
| HN24 | 1.4024 | 1.5342 | 1.0321 | 1.0372 | 1.0511 | 1.5131 | 1.6314 | 1.0299 | 1.0112 | 1.0479 | 1.5926 | 1.6464 | 1.0228 | 1.0183 | 1.0371 |
| ON10 | 2.4080 | 2.4774 | 2.5130 | 2.5025 | 2.4960 | 2.4702 | 2.5408 | 2.5451 | 2.5850 | 2.5225 | 2.5303 | 2.5650 | 2.6413 | 2.6353 | 2.6056 |
| ON18 | 2.4106 | 2.4896 | 2.5124 | 2.5025 | 2.5089 | 2.4700 | 2.5414 | 2.5444 | 2.5363 | 2.5124 | 2.5287 | 2.5648 | 2.6410 | 2.6353 | 2.6033 |
| ON25 | 2.4111 | 2.4802 | 2.5148 | 2.5027 | 2.4992 | 2.4710 | 2.5449 | 2.5449 | 2.5426 | 2.5138 | 2.5300 | 2.5649 | 2.6416 | 2.6353 | 2.6033 |
| NH14 | 1.0155 | 1.0183 | 1.0079 | 1.0120 | 1.0144 | 1.0179 | 1.0086 | 1.0091 | 0.9872 | 1.0141 | 1.0172 | 1.0177 | 1.0073 | 1.0055 | 1.0118 |
| NH28 | 1.0156 | 1.0187 | 1.0081 | 1.0120 | 1.0142 | 1.0179 | 1.0095 | 1.0091 | 0.9899 | 1.0139 | 1.0172 | 1.0177 | 1.0073 | 1.0055 | 1.0118 |
| NH30 | 1.0157 | 1.0183 | 1.0080 | 1.0123 | 1.0129 | 1.0179 | 1.0153 | 1.0091 | 0.9830 | 1.0139 | 1.0171 | 1.0177 | 1.0073 | 1.0055 | 1.0118 |
| CO7 | 1.3183 | 1.3419 | 1.2654 | 1.2650 | 1.2725 | 1.3230 | 1.3382 | 1.2585 | 1.2469 | 1.2670 | 1.3297 | 1.3465 | 1.2584 | 1.2637 | 1.2658 |
| CO15 | 1.3186 | 1.3434 | 1.2652 | 1.2650 | 1.2742 | 1.3230 | 1.3355 | 1.2585 | 1.2434 | 1.2673 | 1.3296 | 1.3465 | 1.2583 | 1.2637 | 1.2660 |
| CO22 | 1.3188 | 1.3426 | 1.2656 | 1.2649 | 1.2734 | 1.3230 | 1.3393 | 1.2584 | 1.2262 | 1.2675 | 1.3299 | 1.3465 | 1.2583 | 1.2637 | 1.2661 |
| CN11 | 1.3058 | 1.3049 | 1.3376 | 1.3384 | 1.3374 | 1.3036 | 1.3015 | 1.3412 | 1.3248 | 1.3390 | 1.2958 | 1.2999 | 1.3336 | 1.3355 | 1.3323 |
| CN19 | 1.3058 | 1.3042 | 1.3376 | 1.3384 | 1.3369 | 1.3036 | 1.3004 | 1.3412 | 1.3418 | 1.3386 | 1.2959 | 1.3000 | 1.3337 | 1.3355 | 1.3323 |
| CN26 | 1.3055 | 1.3049 | 1.6634 | 1.3384 | 1.3369 | 1.3035 | 1.3016 | 1.3413 | 1.3334 | 1.3379 | 1.2957 | 1.3000 | 1.3337 | 1.3355 | 1.3324 |
| OHN9 | 152.40 | 151.05 | 136.37 | 134.30 | 139.98 | 150.83 | 149.30 | 135.08 | 139.49 | 138.23 | 149.66 | 149.22 | 130.01 | 129.35 | 133.68 |
| OHN17 | 152.44 | 151.29 | 136.32 | 135.08 | 139.46 | 150.83 | 149.06 | 135.09 | 136.28 | 138.15 | 149.73 | 149.23 | 129.93 | 129.29 | 133.81 |
| OHN24 | 152.41 | 151.05 | 136.37 | 135.37 | 139.38 | 150.83 | 148.99 | 135.12 | 135.12 | 138.23 | 149.63 | 149.23 | 130.03 | 129.31 | 133.84 |

**Table 2S** Calculated (DFT - B3LYP/6-31+G(d,p); *ab initio* – MP2/6-31+G(d,p)) structural parameters (in Å and °) of the hydrogen bonding in compound **1**

| R=H | E(OH,OH,OH) | | K(OH,OH,NH) | | TS(OH,OH,TS) | | K(OH,NH,NH) | | TS(OH,TS,TS) | | K(NH,NH,NH) | | TS(TS,TS,TS) | |
| --- | --- | --- | --- | --- | --- | --- | --- | --- | --- | --- | --- | --- | --- | --- |
|  | B3LYP | MP2 | B3LYP | MP2 | B3LYP | MP2 | B3LYP | MP2 | B3LYP | MP2 | B3LYP | MP2 | B3LYP | MP2 |
| OH8 | 1.0249 | 1.0020 | 1.0371 | 1.0181 | 1.0279 | 1.0110 | 1.8563 | 1.8577 | 1.0300 | 1.0139 | 1.8639 | 1.8701 | 1.1819 | 1.2091 |
| OH16 | 1.0249 | 1.0080 | 1.0375 | 1.0192 | 1.0283 | 1.0117 | 1.8445 | 1.8519 | 1.1886 | 1.2217 | 1.8664 | 1.8706 | 1.1799 | 1.2075 |
| OH23 | 1.0244 | 1.0080 | 1.8076 | 1.7875 | 1.2019 | 1.2447 | 1.0422 | 1.0226 | 1.1900 | 1.2219 | 1.8639 | 1.8705 | 1.1821 | 1.2097 |
| HN9 | 1.5922 | 1.6466 | 1.5525 | 1.6048 | 1.5816 | 1.6361 | 1.0241 | 1.0198 | 1.5773 | 1.6261 | 1.0228 | 1.0183 | 1.2972 | 1.2572 |
| HN17 | 1.5902 | 1.6463 | 1.5534 | 1.6017 | 1.5839 | 1.6324 | 1.0243 | 1.0197 | 1.2891 | 1.2440 | 1.0228 | 1.0183 | 1.2997 | 1.2587 |
| HN24 | 1.5926 | 1.6464 | 1.0285 | 1.0262 | 1.2743 | 1.2222 | 1.5368 | 1.5859 | 1.2873 | 1.2440 | 1.0231 | 1.0183 | 1.2976 | 1.2565 |
| ON10 | 2.5303 | 2.5650 | 2.5055 | 2.5371 | 2.5242 | 2.5580 | 2.6397 | 2.6330 | 2.5215 | 2.556 | 2.6404 | 2.6357 | 2.4068 | 2.3965 |
| ON18 | 2.5287 | 2.5648 | 2.5071 | 2.5362 | 2.5247 | 2.5570 | 2.6323 | 2.6292 | 2.453 | 2.3953 | 2.6419 | 2.6360 | 2.4072 | 2.3966 |
| ON25 | 2.5300 | 2.5649 | 2.6154 | 2.5992 | 2.4031 | 2.3395 | 2.4966 | 2.5252 | 2.4050 | 2.3954 | 2.6408 | 2.6361 | 2.4072 | 2.3965 |
| CO7 | 1.3297 | 1.3465 | 1.3263 | 1.3405 | 1.3286 | 1.3441 | 1.2583 | 1.2650 | 1.3281 | 1.3424 | 1.2584 | 1.2637 | 1.3052 | 1.3114 |
| CO15 | 1.3296 | 1.3465 | 1.3260 | 1.3396 | 1.3288 | 1.3438 | 1.2581 | 1.2643 | 1.3046 | 1.3109 | 1.2582 | 1.2637 | 1.3054 | 1.3115 |
| CO22 | 1.3299 | 1.3465 | 1.2608 | 1.2705 | 1.3036 | 1.3101 | 1.3262 | 1.3385 | 1.3045 | 1.3110 | 1.2584 | 1.2637 | 1.3052 | 1.3113 |
| CN11 | 1.2958 | 1.2999 | 1.2991 | 1.3032 | 1.2971 | 1.3017 | 1.3321 | 1.3334 | 1.2979 | 1.3024 | 1.3337 | 1.3355 | 1.3075 | 1.3113 |
| CN19 | 1.2959 | 1.3000 | 1.2996 | 1.3032 | 1.2971 | 1.3013 | 1.3327 | 1.3350 | 1.3069 | 1.3103 | 1.3337 | 1.3355 | 1.3073 | 1.3112 |
| CN26 | 1.2957 | 1.3000 | 1.3283 | 1.3281 | 1.3065 | 1.3098 | 1.3007 | 1.3042 | 1.3072 | 1.3110 | 1.3336 | 1.3355 | 1.0374 | 1.3113 |
| OHN | 149.656 | 149.22 | 150.11 | 149.83 | 152.08 | 149.26 | 130.58 | 130.12 | 149.82 | 149.58 | 130.01 | 129.30 | 152.21 | 152.69 |

**Scheme S1** Energy scheme of tautomers (black columns) and transition states (grey columns) for compound **3** calculated at the B3LYP/6-31+G(d,p) levels of theory.

**Fig. 1S** Details of time evolution of the interatomic distances in the hydrogen bridges of compound **1**. CPMD simulation results. Different colors denote three various hydrogen bridges. Upper curves in each graph: d(OH) (Å), lower curves: d(NH) (Å)

**
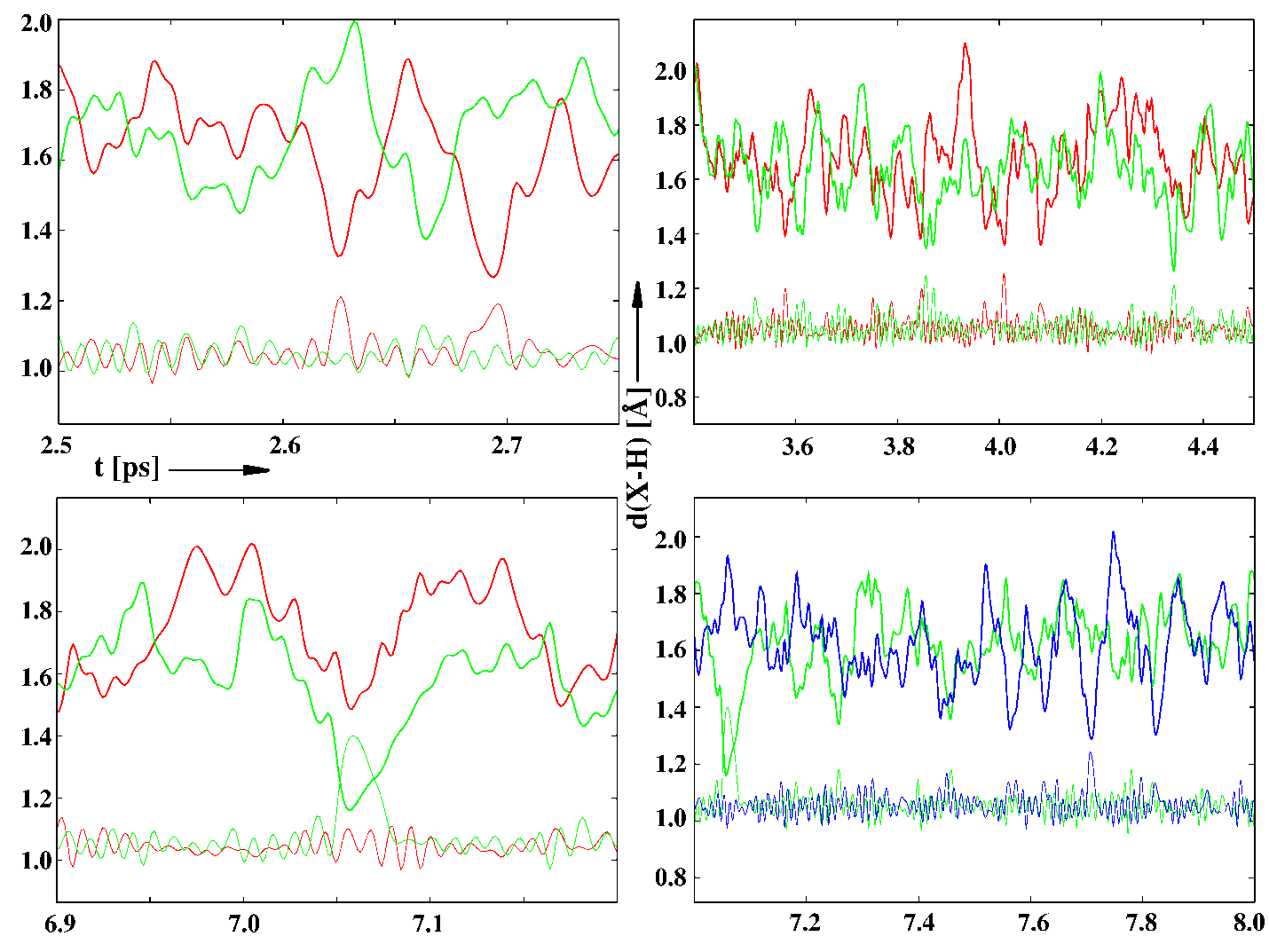
**
